# Covid-19 vaccine effectiveness against post-covid-19 condition among 589 722 individuals in Sweden: population based cohort study

**DOI:** 10.1136/bmj-2023-076990

**Published:** 2023-11-22

**Authors:** Lisa Lundberg-Morris, Susannah Leach, Yiyi Xu, Jari Martikainen, Ailiana Santosa, Magnus Gisslén, Huiqi Li, Fredrik Nyberg, Maria Bygdell

**Affiliations:** 1 Department of Microbiology and Immunology, Institute of Biomedicine, Sahlgrenska Academy, University of Gothenburg, Gothenburg, Sweden; 2 Region Västra Götaland, Department of Clinical Pharmacology, Sahlgrenska University Hospital, Gothenburg, Sweden; 3 AstraZeneca, Mölndal, Sweden; 4 School of Public Health and Community Medicine, Institute of Medicine, Sahlgrenska Academy, University of Gothenburg, Gothenburg, Sweden; 5 Bioinformatics and Data Centre, Sahlgrenska Academy, University of Gothenburg, Gothenburg, Sweden; 6 Department of Infectious Diseases, Institute of Biomedicine, Sahlgrenska Academy, University of Gothenburg, Gothenburg, Sweden; 7 Region Västra Götaland, Department of Infectious Diseases, Sahlgrenska University Hospital, Gothenburg, Sweden; 8 Department of Internal Medicine and Clinical Nutrition, Institute of Medicine, Sahlgrenska Academy, University of Gothenburg, Gothenburg, Sweden

## Abstract

**Objective:**

To investigate the effectiveness of primary covid-19 vaccination (first two doses and first booster dose within the recommended schedule) against post-covid-19 condition (PCC).

**Design:**

Population based cohort study.

**Setting:**

Swedish Covid-19 Investigation for Future Insights—a Population Epidemiology Approach using Register Linkage (SCIFI-PEARL) project, a register based cohort study in Sweden.

**Participants:**

All adults (≥18 years) with covid-19 first registered between 27 December 2020 and 9 February 2022 (n=589 722) in the two largest regions of Sweden. Individuals were followed from a first infection until death, emigration, vaccination, reinfection, a PCC diagnosis (ICD-10 diagnosis code U09.9), or end of follow-up (30 November 2022), whichever came first. Individuals who had received at least one dose of a covid-19 vaccine before infection were considered vaccinated.

**Main outcome measure:**

The primary outcome was a clinical diagnosis of PCC. Vaccine effectiveness against PCC was estimated using Cox regressions adjusted for age, sex, comorbidities (diabetes and cardiovascular, respiratory, and psychiatric disease), number of healthcare contacts during 2019, socioeconomic factors, and dominant virus variant at time of infection.

**Results:**

Of 299 692 vaccinated individuals with covid-19, 1201 (0.4%) had a diagnosis of PCC during follow-up, compared with 4118 (1.4%) of 290 030 unvaccinated individuals. Covid-19 vaccination with any number of doses before infection was associated with a reduced risk of PCC (adjusted hazard ratio 0.42, 95% confidence interval 0.38 to 0.46), with a vaccine effectiveness of 58%. Of the vaccinated individuals, 21 111 received one dose only, 205 650 received two doses, and 72 931 received three or more doses. Vaccine effectiveness against PCC for one dose, two doses, and three or more doses was 21%, 59%, and 73%, respectively.

**Conclusions:**

The results of this study suggest a strong association between covid-19 vaccination before infection and reduced risk of receiving a diagnosis of PCC. The findings highlight the importance of primary vaccination against covid-19 to reduce the population burden of PCC.

## Introduction

A global pandemic due to covid-19 was declared in March 2020, and by June 2023 just over 767 million covid-19 cases, including 6.9 million deaths, had been reported to the World Health Organization.^1^ Effective vaccines against covid-19 were rapidly developed, and the first vaccine dose in Sweden was administered in December 2020, less than one year after the start of the pandemic.^2^ The efficacy and effectiveness of covid-19 vaccines against SARS-CoV-2 infection and severe manifestations of acute covid-19 have been shown.^3^ ^4^ ^5^ ^6^ Shortly after the start of the pandemic, reports emerged describing persistent symptoms among some people who had recovered from covid-19, regardless of whether they had been admitted to hospital with the disease, often referred to as long covid or post-covid-19 condition (PCC). We previously found that 2% of adults with covid-19 in Sweden received a diagnosis of PCC, although studies relying on self-reported persistent symptoms usually report a higher incidence rate. A recent umbrella review showed that the prevalence of prolonged symptoms of covid-19 varied between 2% and 53% among different study populations.^7^ Generally described symptoms of PCC include fatigue, dyspnoea, cognitive impairment, headache, muscle pain, and cardiac abnormalities such as chest pain and palpitations.^8^ Furthermore, by using machine learning algorithms a recent study identified four clinical phenotype clusters within the PCC group; chronic fatigue-like syndrome, respiratory syndrome, chronic pain syndrome, and neurosensorial syndrome.^9^ According to a Delphi consensus held by WHO, PCC can occur “in individuals with a history of probable or confirmed SARS-CoV-2 infection, usually three months from the onset of covid-19 with symptoms that last for at least two months and cannot be explained by an alternative diagnosis.”^10^ Few studies have evaluated the effectiveness of covid-19 vaccines to prevent PCC in large population based settings. A recent systematic review concluded that receiving a covid-19 vaccine before SARS-CoV-2 infection had a protective effect against PCC in 10 of the 12 included studies, with effect estimates (odds ratios and hazard ratios) ranging from 0.48 to 0.87 for any vaccine dose before infection.^11^ The authors did not, however, conduct a meta-analysis because of the high heterogeneity in the methodology and data between studies. They concluded that owing to inadequate adjustment for confounders and risk of bias in the included studies, the certainty of evidence was low.

Sweden has a long history of collecting health and demographic data from its population in national registers. By using an individual’s personal identification number as the unique identifier in multiple registers, it is possible to link information between different data sources with high linkage quality.^12^ In Sweden, the ICD-10 (international classification of diseases, 10th revision) diagnosis code for PCC (U09.9) had already been implemented in October 2020. Since then the National Board of Health and Welfare has encouraged the use of the diagnosis code for conditions related to previous covid-19 and describes it as: “An additional code that can be used to describe a condition’s association with covid-19.” During the pandemic, the National Board of Health and Welfare continuously released reports describing the prevalence of PCC using the diagnosis code from specialist healthcare and aggregated level data from primary healthcare.^13^ ^14^ ^15^ We have recently shown that most patients (>85%) with a PCC diagnosis in Sweden received their diagnosis in primary healthcare.^16^ Hence the use of individual level primary healthcare data is vital when studying PCC in Sweden. Through the SCIFI-PEARL (Swedish Covid-19 Investigation for Future Insights—a Population Epidemiology Approach using Register Linkage) project database, we had access to national register data on vaccination status, diseases, sociodemographic information, and primary healthcare data from the two largest regions of Sweden (covering about 40% of the Swedish population). We investigated the effectiveness of primary covid-19 vaccination (the first two vaccine doses and the first booster dose within the recommended schedule) against PCC among individuals vaccinated before infection using real world data.

## Methods

### Study design and data sources

This population based cohort study is part of the project SCIFI-PEARL, a nationwide linked multiregister, observational study of the covid-19 pandemic in Sweden.^17^ Because primary healthcare data are important when studying PCC in Sweden,^16^ we used register data for all adult (≥18 years) residents in Region Stockholm and Region Västra Götaland (Sweden’s two largest regions, covering about 40% of the Swedish population^18^), from which we had access to information from primary healthcare. By using unique personal identification numbers, we retrieved data on administered covid-19 vaccines for each individual from the National Vaccination Register; positive SARS-CoV-2 polymerase chain reaction (PCR) results from the National Register of Notifiable Diseases; ICD-10 diagnosis codes for covid-19, PCC, and comorbidities; and number of healthcare contacts during 2019 for inpatient and outpatient specialist care from the National Patient Register and for primary care from two regional databases of all public and most private primary healthcare (VAL and VEGA, in Region Stockholm and Region Västra Götaland, respectively); as well as death, emigration, demographic, and socioeconomic data from the Longitudinal Integrated Database for Health Insurance and Labour Market Studies of Statistics Sweden. We used the Swedish Intensive Care Register together with the inpatient part of the National Patient Register to identify patients with covid-19 who were admitted to hospital, including those treated in the intensive care unit (ICU).

### Study population and follow-up

We included individuals with covid-19 first registered during the study inclusion period, defined as between 27 December 2020 (when vaccination started in Sweden^2^) and 9 February 2022 (the end of full population PCR testing in Sweden^19^). This period was selected to include the time during which simultaneous vaccination and PCR testing occurred in Sweden. Covid-19 was defined as a positive SARS-CoV-2 PCR test result registered in the National Register of Notifiable Diseases, or an ICD-10 diagnosis code (U07.1 or U07.2 as a main or secondary diagnosis) registered in the National Patient Register, VAL, VEGA, or the Swedish Intensive Care Register. The covid-19 index date represents the first registration of covid-19 in any of these registers. Included individuals were then followed from 28 days after the covid-19 index date until PCC diagnosis, vaccination, reinfection, death, emigration, or end of follow-up (30 November 2022), whichever came first. The inclusion criteria were to be alive and living in Region Stockholm or Västra Götaland, Sweden, at the start of follow-up, and not to have been vaccinated after the covid-19 index date to start of follow-up. Thus we excluded those individuals who had been vaccinated, emigrated, or died within 28 days after infection. We regarded an additional registered SARS-CoV-2 infection ≥90 days after the covid-19 index date as reinfection.

### Vaccination and outcome

In Sweden, the covid-19 vaccination programme started on 27 December 2020 and was implemented in four consecutive stages; stages 1-3 (older age groups (≥60 years), healthcare or elderly care workers, and various risk groups) and stage 4 (younger age groups who had not been part of the previous stages), with stage 4 initiated in most parts of Sweden by May 2021.^20^ In the present study, at least one administered dose of any of the available covid-19 vaccines in Sweden before the covid-19 index date was defined as having received a vaccine. We studied the primary vaccination series—that is, the first two doses and the first booster dose within the recommended schedule. During the study period, the available vaccines in Sweden included BNT162b2 (Pfizer-BioNTech), mRNA-1273 (Moderna), AZD1222 (Oxford-AstraZeneca), Ad26.COV2.S (Janssen/Johnson & Johnson), and NVX-CoV2373 (Novavax).^21^ To minimise the risk of including double registrations of one vaccine dose, we required a minimum number of days between two registered doses. Based on vaccination guidelines and the type of vaccine previously received, a requirement of at least 19 days was set for BNT162b2 and at least 25 days for AZD1222 and mRNA-1273.^22^ We classified vaccines by dose (≥1 dose, as well as subdivided into one dose, two doses, and three or more doses). The primary outcome was a clinical diagnosis of PCC, defined as ICD-10 code U09.9 in the National Patient Register, VEGA, or VAL as the main or secondary diagnosis ≥28 days after the covid-19 index date. A minimum of 28 days between the covid-19 index date and PCC diagnosis was required, as a PCC diagnosis within the 28 days was interpreted as a likley misclassification relating to acute infection rather than PCC.

### Covariates

We obtained information on age, sex, education level, employment status, country of birth, emigration, and date of death from the Longitudinal Integrated Database for Health Insurance and Labour Market Studies database. Age was defined at study start (27 December 2020) and categorised into five groups (18-34, 35-44, 45-54, 55-64, and ≥65 years). Level of education was divided into four categories: primary school (<10 years), secondary school (10-12 years), tertiary school (>12 years), and unknown. Employment status was categorised as employed, unemployed, or unknown. Countries of birth were merged into continental regions, including Asia and Oceania; Africa; Europe, except for Sweden; North and South America; and unknown. The included comorbidities have been shown to relate to vaccination status and PCC diagnosis^16^ ^23^ ^24^ and comprised broad categories of diabetes (ICD-10: E10-E11), cardiovascular disease (ICD-10: I00-I99), respiratory disease (ICD-10: J40-J99), and psychiatric disease (ICD-10: F00-F99) as main and secondary diagnoses from inpatient and outpatient specialist care in the National Patient Register and from primary healthcare in VEGA and VAL, 1 January 2015 to 31 December 2019. We categorised the severity of acute covid-19 as hospital admission (treated or not treated in an ICU) or no hospital admission, using main and secondary diagnoses of covid-19 within 28 days after the covid-19 index date in the Swedish Intensive Care Register and the inpatient part of the National Patient Register. The number of healthcare contacts during 2019 was categorised as 0, 1-3, 4-10, >10, or unknown. We defined a healthcare contact as any registered contact (including by telephone) with primary healthcare, specialist outpatient care, and specialist inpatient care. If more than one contact was registered on the same day, it was counted as one contact only. For inpatient care, we used the date of admission, with every inpatient period counted as one contact. Study individuals were also classified according to the covid-19 variant of concern that predominated during their covid-19 index date. In Sweden, pre-alpha variants predominated roughly from February 2020 to January 2021, followed by alpha (February 2021 to June 2021), delta (July 2021 to December 2021), and omicron (January 2022 to end of study inclusion).^25^ Owing to the low numbers of people with covid-19 in the period when the pre-alpha variants were predominant, for the purpose of analysis we combined the periods when the pre-alpha variants and alpha were predominant.

### Statistical analyses

Descriptive statistics are presented as number and percentage, or as median and interquartile range (IQR). We tested for significance between the groups using the χ^2^ test and the Mann-Whitney U test. In addition, to be able to further assess the distribution of baseline variables of different types and magnitude or prevalence between the unvaccinated and vaccinated groups, we used the standardised mean difference, with larger values indicating larger differences (greater imbalance) between the groups, and values ≤0.1 indicating good balance.

We performed Cox proportional hazards regressions to estimate the effectiveness of covid-19 vaccination before infection and risk of developing PCC. The assumption of proportional hazards was fulfilled as assessed by visual evaluation of a Schoenfeld residual plot of the vaccination status. We included three models: a crude model with no adjustments; a partially adjusted model with adjustments for age, sex, and predominant variant at the time of infection; and a fully adjusted model with the same adjustments as for the partially adjusted model in addition to comorbidities (diabetes and cardiovascular, respiratory, and psychiatric disease), number of healthcare contacts during 2019, education level, employment status, and region of birth. The results are presented as hazard ratios or adjusted hazard ratios (corresponding to the fully adjusted model) along with corresponding 95% confidence intervals. Vaccine effectiveness was calculated as 100×(1−hazard ratio). In the main analysis, at least one vaccine dose (any dose) before the covid-19 index date was defined as having been vaccinated. Separate analyses were also performed with vaccination stratified into one dose, two doses, or three or more doses before the covid-19 index date. The Kaplan-Meier method was used to estimate cumulative incidence curves of PCC in the unvaccinated and vaccinated groups. In exploratory analyses, we further investigated a possible pathway for the potential protective effect of the covid-19 vaccines against PCC by adding severity of the acute infection into the regression model as a mediating factor. We also stratified by severity of the acute infection. All analyses were performed using R Statistical Software (version 4.2.2; R Core Team 2023).

#### Subgroup and sensitivity analyses

Analyses to evaluate effect modification were performed by including an interaction term between the vaccination variable (any vaccine before covid-19) and each adjustment variable (one interaction term at a time). We regarded an interaction as statistically significant if the P value of the interaction term was <0.05. Further stratification was planned for the three most relevant variables if they showed significant interaction terms: sex, age group, and predominant variant at the time of the covid-19 index date. Stratification was also done for comorbidities and for the median time between vaccination and the covid-19 index date (vaccination ≥126 days before covid-19 index date, and <126 days). Separate subgroup analyses for the different vaccines were also perfomed, categorised by the combination of vaccines used for the first two doses before infection. These analyses were restricted to the period 3 February 2021 to 16 August 2021 (when the first dose of the three most common vaccines in Sweden was administered: BNT162b2, mRNA-1273, and AZD1222) (see supplementary figure S1).

Three sensitivity analyses were also performed. Firstly, we restricted the vaccinated population to those who had received their last vaccine dose more than 14 days before the covid-19 index date. Secondly, we required at least 90 (instead of 28) days between the covid-19 index date and a diagnosis of PCC. Finally, we restricted the vaccinated population to those who had received two or three doses before the covid-19 index date.

### Patient and public involvement

No patients or members of the public were directly involved in this research. A patient reviewer did, however, provide insightful comments during the review process. 

## Results

### Descriptive statistics

**Fig 1:**
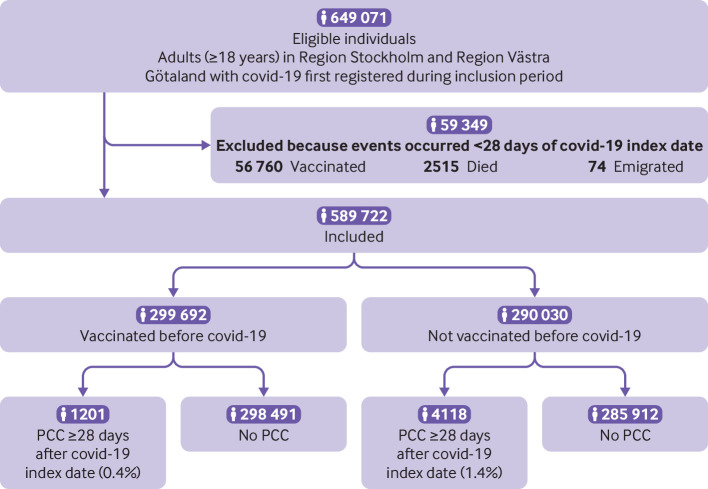
Flowchart of study population with covid-19 during study inclusion period 27 December 2020 to 9 February 2022. PCC=post-covid-19 condition

During the study inclusion period, 649 071 individuals in Sweden’s two largest regions were registered as having covid-19 for the first time. Overall, 59 349 individuals were excluded: 56 760 were vaccinated, 2515 died, and 74 emigrated within 28 days of the covid-19 index date. In total, 589 722 individuals fulfilled the inclusion criteria to participate in the study (fig 1).

Of the 589 722 individuals in the study population, 299 692 (50.8%) had received at least one dose of a covid-19 vaccine before the covid-19 index date, and 290 030 (49.2%) had not been vaccinated at the time of infection (table 1). Of the vaccinated individuals, 21 111 received one dose only, 205 650 received two doses, 72 843 received three doses, and 88 received more than three doses before the covid-19 index date (table 1). BNT162b2 was most commonly used for the first two doses (see supplementary table S1). The median time from last vaccination to the covid-19 index date was 126 days (IQR 47-160 days). More women than men had been vaccinated before covid-19 (56.7% *v* 44.3%, P<0.001), and the median age among those vaccinated was significantly higher than among those not vaccinated (42 years (IQR 32-53) *v* 39 years (29-50), P<0.001) (table 1). Although most of the study population was not admitted to hospital with covid-19, unvaccinated individuals were more likely to be admitted than vaccinated individuals (4.0% *v* 1.5%, P<0.001) (table 1). In the unvaccinated group, 174 689 individuals (60.2%) had a covid-19 index date during the period when the alpha variant was predominant, whereas individuals in the vaccinated group were mostly infected during the omicron era (n=224 330, 74.9%) (table 1).

**Table 1:** Descriptive statistics of study population, stratified according to vaccination status before covid-19 infection. Values are number (percentage) unless stated otherwise

	Vaccination status pre-covid-19		Standardised mean difference
	Not vaccinated (n=290 030)	Vaccinated (n=299 692)	
Sex:			0.10
Men	142 921 (49.3)	132 650 (44.3)	
Women	147 109 (50.7)	167 042 (56.7)	
Median (IQR) age (years)*	39 (29-50)	42 (32-53)	0.20
Age group* (years):			0.19
18-34	118 365 (40.8)	99 664 (33.3)	
35-44	66 840 (23.0)	73 285 (24.5)	
45-54	55 279 (19.1)	60 790 (20.3)	
55-64	32 001 (11.0)	37 324 (12.5)	
≥65	17 545 (6.0)	28 629 (9.6)	
Region of birth:			0.23
Africa	10 121 (3.5)	6498 (2.2)	
Asia and Oceania	37 697 (13.0)	26 980 (9.0)	
Europe†	21 493 (7.4)	15 323 (5.1)	
North and South America	6683 (2.3)	6269 (2.1)	
Sweden	196 490 (67.7)	232 860 (77.7)	
Unknown	17 546 (6.0)	11 762 (3.9)	
Education:			0.23
Primary school (<10 years)	44 093 (15.2)	31 835 (10.6)	
Secondary school (10-12 years)	118 613 (40.9)	108 470 (36.2)	
Tertiary school (>12 years)	116 232 (40.1)	152 126 (50.8)	
Unknown	11 092 (3.8)	7261 (2.4)	
Employment status:			0.05
Employed	248 127 (85.6)	260 318 (86.9)	
Unemployed	37 847 (13.0)	36 621 (12.2)	
Unknown	4056 (1.4)	2753 (0.9)	
Comorbidities‡:			
Diabetes	9388 (3.2)	11 449 (3.8)	0.03
Cardiovascular disease	42 684 (14.7)	54 491 (18.2)	0.09
Respiratory disease	21 807 (7.5)	25 055 (8.4)	0.03
Psychiatric disease	81 807 (28.2)	86 841 (29.0)	0.02
No of healthcare contacts in 2019:			0.07
0	63 866 (22.0)	60 305 (20.1)	
1-3	85 699 (29.5)	87 623 (29.2)	
4-10	76 135 (26.3)	81 056 (27.0)	
>10	51 609 (17.8)	58 983 (19.7)	
Unknown	12 721 (4.4)	11 725 (3.9)	
No of vaccine doses before covid-19:			
1	-	21 111 (7.0)	
2	-	205 650 (68.6)	
3	-	72 843 (24.3)	
4	-	83 (<0.1)	
5	-	5 (<0.1)	
Severity of covid-19:			0.15
Admitted to hospital, ICU	389 (0.1)	77 (<0.1)	
Admitted to hospital, not ICU	11 178 (3.9)	4610 (1.5)	
Not admitted to hospital	278 463 (96.0)	295 005 (98.4)	
Predominant variant during acute infection§:			2.4
Pre-alpha	53 021 (18.3)	405 (0.1)	
Alpha	174 689 (60.2)	10 394 (3.5)	
Delta	26 260 (9.1)	64 563 (21.5)	
Omicron	36 060 (12.4)	224 330 (74.9)	

ICD-10=international classification of diseases, 10th revision;.ICU=intensive care unit; IQR=interquartile range.

Study population included all adult (≥18 years) residents in the two largest regions of Sweden with covid-19 first registered during the study inclusion period, 27 December 2020 to 9 February 2022.

* Age at study start, 27 December 2020.

† Not including Sweden.

‡ Comorbidities registered with ICD-10 diagnosis codes in specialist inpatient and outpatient healthcare registers and regional primary healthcare databases during 2015-19: diabetes (E10-E11), cardiovascular disease (I00-I99), respiratory disease (J40-J99), and psychiatric disease (F00-F99).

§ In Sweden, pre-alpha variants predominated from February 2020 to January 2021, followed by alpha from February 2021 to June 2021, delta from July 2021 to December 2021, and omicron from January 2022 and until end of inclusion 9 February 2022.

**Fig 2:**
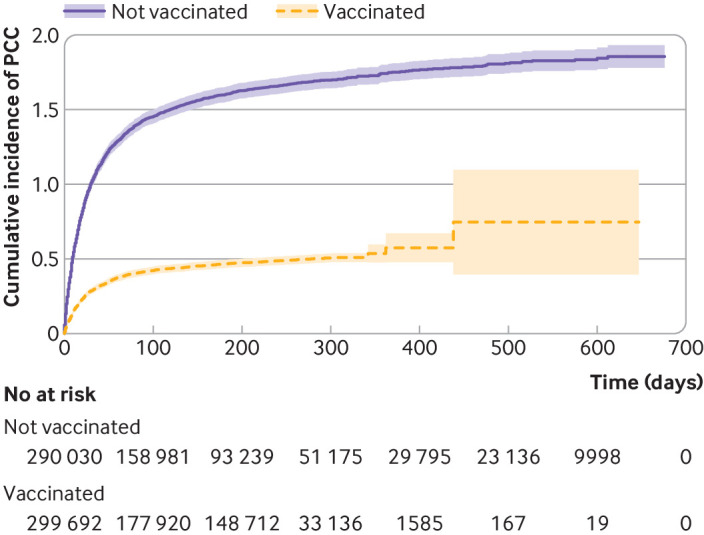
Cumulative incidence of PCC, using Kaplan-Meier failure function, for individuals vaccinated or not vaccinated against covid-19. Study population included all adult (≥18 years) residents in the two largest regions of Sweden with covid-19 first registered during the study inclusion period, 27 December 2020 to 9 February 2022. PCC=post-covid-19 condition

Median follow-up from 28 days after the covid-19 index date was 129 (IQR 51-287) days in the total study population (vaccinated: 197 (IQR 42-288) days, not vaccinated: 112 (IQR 57-282) days). Median follow-up until a PCC diagnosis was similar between the vaccinated and unvaccinated groups (18 (IQR 7-47) days *v* 17 (IQR 6-39) days). Individuals who received three or more vaccine doses before infection had the longest follow-up (median 250 (IQR 126-283) days), whereas those who had only received one dose before infection had the shortest follow-up (median 42 (IQR 11-281) days). For both vaccinated and unvaccinated individuals, the most common reason for censoring during follow-up was vaccination, followed by end of follow-up (see supplementary table S2). Captured reinfections were rare during the study period (n=12 888, 2.2%) and significantly more common in the unvaccinated group than vaccinated group (3.3% *v* 1.1%, P<0.001) (see supplementary table S2). Among vaccinated individuals, 1201 (0.4%) had a diagnosis of PCC during follow-up, compared with 4118 (1.4%) among unvaccinated individuals (fig 2 and supplementary table S2).

### Vaccine effectiveness

Receiving at least one dose of a covid-19 vaccine before the covid-19 index date was associated with a reduced risk of PCC (adjusted hazard ratio 0.42 (95% confidence interval 0.38 to 0.46), with a vaccine effectiveness of 58% (table 2). Vaccine effectiveness increased with increasing number of vaccine doses before covid-19. Adjusted hazard ratios for one dose, two doses, and three doses were 0.79 (0.68 to 0.91), 0.41 (0.37 to 0.45), and 0.27 (0.23 to 0.32), with a respective vaccine effectiveness of 21%, 59%, and 73% (table 2).

**Table 2:** Vaccine effectiveness and hazard ratios, with 95% confidence intervals, between covid-19 vaccination before infection and a diagnosis of post-covid-19 condition, overall and in separate analyses stratified by number of vaccine doses

	Total No	No (%) with PCC	Hazard ratio (95% CI)			P value‡	Vaccine effectiveness % (95% CI)‡
			Crude	Partially adjusted model*	Fully adjusted model†		
No vaccination	290 030	4118 (1.4)	Reference	Reference	Reference	Reference	Reference
Any vaccination§	299 692	1201 (0.4)	0.29 (0.27 to 0.31)	0.41 (0.38 to 0.45)	0.42 (0.38 to 0.46)	<0.001	58 (54 to 62)
**Separate stratified analyses**							
1 dose¶	21 111	192 (0.9)	0.83 (0.72 to 0.96)	0.79 (0.68 to 0.91)	0.79 (0.68 to 0.91)	0.002	21 (9 to 32)
2 doses¶	205 650	743 (0.4)	0.27 (0.25 to 0.29)	0.40 (0.36 to 0.45)	0.41 (0.37 to 0.45)	<0.001	59 (55 to 63)
≥3 doses¶	72 931	266 (0.4)	0.23 (0.20 to 0.26)	0.26 (0.22 to 0.31)	0.27 (0.23 to 0.32)	<0.001	73 (68 to 77)

CI=confidence interval; PCC=post-covid-19 condition.

Study population included all adult (≥18 years) residents in the two largest regions of Sweden with covid-19 first registered during the study inclusion period, 27 December 2020 to 9 February 2022.

* Cox proportional hazards regression model adjusted for age, sex, and predominant virus variant at the time of infection.

† Cox proportional hazards regression model adjusted for age, sex, predominant virus variant at the time of infection, comorbidities (diabetes and cardiovascular, respiratory, and psychiatric disease), number of healthcare contacts during 2019, region of birth, education level, and employment status.

‡ Based on fully adjusted model.

§ Including 1-5 doses.

¶ Analysis performed versus no vaccination.

To evaluate potential vaccine effectiveness against PCC through the reduced risk of hospital admission for acute infection, we added severity of the acute infection (hospital admission requiring ICU admission, hospital admission not requiring ICU admission, no hospital admission) to the regression model. The adjusted hazard ratio for any dose before infection was 0.54 (0.50 to 0.60) and for one dose, two doses, and three or more doses was 0.81 (0.70 to 0.93), 0.53 (0.48 to 0.59), and 0.42 (0.35 to 0.49), respectively (see supplementary table S3). We then stratified the analysis of any dose by severity of the acute infection, resulting in similar adjusted hazard ratios between the hospital admission without ICU admission group and the no hospital admission group (0.57 (0.48 to 0.68) *v* 0.56 (0.50 to 0.62), respectively) (see supplementary table S4).

### Subgroup and sensitivity analyses

In the subgroup analysis stratifying on the median time between last vaccination and infection (126 days), vaccine effectiveness against PCC for any dose before covid-19 was slightly lower for longer duration since last dose and slightly higher for shorter duration compared with the main analysis (≥126 days: 49%, <126 days: 63%) (see supplementary table S5). Individuals with a last vaccination more than 126 days from infection mainly received two doses (0.1% had received ≥3 doses, data not shown). The interaction terms for age, sex, predominant variant at the time of the covid-19 index date, employment status, number of healthcare contacts, diabetes, and cardiovascular disease were significant when separately added to the full model. Men showed a higher vaccine effectiveness than women (≥1 dose: 64% and 54%, respectively). The highest vaccine effectiveness by age group was shown in those aged 55-64 years (≥1 dose: 69%) and the lowest in those aged 18-34 years (≥1 dose: 28%). We also stratified infection on the preodominant variant and found that vaccine effectiveness was highest in individuals with covid-19 during the delta period (68%) (see supplementary table S4). No major differences were found in the analyses stratified by combinations of vaccines for the first two doses before covid-19, with adjusted hazard ratios ranging from 0.43 (0.32 to 0.58) to 0.49 (0.44 to 0.54); except for the combination of AZD1222 and BNT162b2 (see supplementary table S6).

In the first sensitivity analysis, we restricted the vaccinated group to those individuals who were vaccinated at least 14 days before the covid-19 index date (n=265 299, 88.5% of the total vaccinated group), which only marginally changed the results compared with the main analyses (see supplementary table S7). In the second sensitivity analysis, we restricted to a minimum of 90 days between the covid-19 index date and PCC diagnosis, which yielded similar results to the analysis with a requirement of 28 days (see supplementary table S8). Lastly, when we restricted the vaccinated group to those who only received two or three doses before infection, vaccine effectiveness was 64% (see supplementary table S9).

## Discussion

In this large register based cohort study including 589 722 residents from the two largest regions of Sweden, we found a strong association between vaccination before first registered covid-19 and a reduced risk of receiving a diagnosis of PCC. In the study population, unvaccinated individuals had an almost fourfold higher proportion of PCC diagnoses compared with those who were vaccinated before infection (1.4% *v* 0.4%). We found a vaccine effectiveness against PCC of 58% for any dose within the primary vaccination series (ie, the first two doses and the first booster dose administered within the recommended schedule) given before a first registered infection. Vaccine effectiveness increased with each dose in the series: 21% for one dose, 59% for two doses, and 73% for three or more doses.

### Findings in context

The few earlier studies on vaccine effectiveness against the long term effects of covid-19 have mostly shown protective effects, with a wide range of effect estimates,^26^ ^27^ but some failed to show an overall protective effect.^28^ ^29^ The methodology and data included in the earlier studies were heterogeneous and had limitations. Study populations have rarely been population based and often have included a small number of participants.^30^ ^31^ Analyses of different effects for different numbers of vaccine doses before covid-19 have not always been performed.^27^ ^32^ Because vaccination during follow-up has often not been a criterion for censoring, both vaccinated and unvaccinated individuals have been included in the unvaccinated group. In the present study, we used population based survival data of 589 722 individuals, censoring at both vaccination and reinfection, and report vaccine effectiveness separately for any dose, one dose, two doses, and three or more doses. Earlier studies have generally lacked a clear definition of PCC, and symptoms have often been self-reported,^29^ ^30^ ^31^ ^33^ whereas we used register based clinical diagnoses of PCC as the outcome. Furthermore, in earlier studies follow-up duration has often been short,^29^ whereas in our study the median follow-up was 129 days from 28 days after a first registered infection. A recent systematic review concluded that being vaccinated against covid-19 before infection had a protective effect on PCC in 10 of the 12 included studies, with effect estimates ranging from 0.48 to 0.87 for any vaccine dose given before infection.^11^ Owing to high heterogeneity between studies and the low certainty of evidence, no meta-analysis was performed. In other systematic reviews, meta-analyses have, however, included several of these studies, but the results should be interpreted with caution.^34^ ^35^ One of these meta-analyses concluded that receiving two doses of vaccine before covid-19 was associated with a lower risk of PCC compared with no vaccination, with an odds ratio of 0.64,^34^ and that the odds ratio was 0.71 with at least one dose before infection.^35^

Using register data from the whole adult population in the two largest regions of Sweden, we showed that vaccination against covid-19 before infection was associated with a decreased risk of receiving a diagnosis of PCC. When stratifying by the median time between last vaccination and infection (126 days) to assess the potential different effects of recent versus earlier vaccination, we found that receiving the last vaccine dose more than 126 days before covid-19 was still associated with a relatively high vaccine effectiveness against PCC, and only slightly lower than in the main analysis. In addition, to ensure sufficient time between vaccination and the acute infection, in a sensitivity analysis we restricted the vaccinated population to those who received their last vaccine dose more than 14 days before covid-19, and the estimated vaccine effect did not markedly change from the main analysis. Furthermore, in the main analyses we only considered the first PCC diagnosis at least 28 days from infection, but in sensitivity analyses we required at least 90 days from infection, with similar results.

Studies have shown that women may develop greater immune responses to vaccination than men,^36^ although this does not necessarily translate to better protection against the disease. In our study, men showed a higher vaccine effectiveness against PCC than women. It has not yet been fully established whether PCC is more likely to occur with particular variants. Available data suggest, however, that individuals infected with the omicron variant are at lower risk of developing long term effects of covid-19 than individuals infected with the other variants.^37^ ^38^ ^39^ Nonetheless, it is difficult to determine if this lower risk is associated with the specific variant or is the result of immunity from previous infections or vaccinations, or as a result of shorter follow-up durations. A small study evaluating the protective effect of covid-19 vaccines against PCC, which included individuals with infections during the omicron period as well as the earlier periods, did not show significantly different results between the variants.^30^ In the present study, the study population included individuals with infections at the time when the alpha and delta variants predominated, and also during part of the pre-alpha and omicron periods. Although vaccination coverage was not evenly distributed during these periods, stratifying on the period of dominant variant at the time of infection showed only a slightly lower vaccine effectiveness against PCC in the omicron period than in the pre-alpha and alpha periods. As we did not have access to virus sequencing data in our analysis, we used the time of infection as a proxy for variant. Consequently, the variant causing acute infection in some of the study individuals might have been misclassified.

The pathogenesis of PCC has not yet been clarified, but several mechanisms have been proposed relating to the different symptom manifestations and it has become increasingly evident that patients with PCC are a heterogenous group. Potential mechanisms include organ damage, abnormal immune activation during acute infection, reactivation of other viruses, altered systemic immunity, autoimmunity, and sustained immune activation due to viral persistence.^40^ Determining the pathogenesis might suggest potential pathways for the protective effect of the vaccines—for example, a reduced viral load during the acute infection after vaccination could reduce the virus’s persistence with lasting immune activation. Different symptom clusters of PCC may have different pathogeneses and therefore different mechanisms for the vaccine effect. We have shown that almost 37% of patients with covid-19 treated in the ICU subsequently have a PCC diagnosis.^16^ Covid-19 vaccines have been shown to protect against hospital admission with covid-19,^41^ which could be one pathway for the vaccines to exert a protective effect against PCC. In our analysis, vaccine effectiveness against PCC seemed to be only partly explained by a decreased risk of hospital admission. In addition, analyses stratified on severity of acute disease as indicated by the need for hospital admission showed that vaccine effectiveness was similar in both the group admitted to hospital without ICU admission and the group with no hospital admission. Furthermore, a study showed that those who were vaccinated after covid-19 had a lower risk of developing PCC compared with those who were unvaccinated 12 weeks after covid-19.^26^ This, together with the findings in the present study, support the hypothesis of pathways beyond the protective effect against hospital admission that may contribute to the protective effect of covid-19 vaccines against PCC. It is also important to note that symptoms of PCC are frequently observed not only in patients with confirmed covid-19 but also in those without a positive SARS-CoV-2 PCR test result.^42^

### Strengths and limitations of this study

The present study has several strengths. Firstly, we used register based data collected from high quality registers, resulting in essentially no loss to follow-up and a low risk of self-reporting bias. In Sweden, it is mandatory and regulated by law to register every administered covid-19 vaccine dose in the national vaccination register. Therefore the exposure data (vaccination) are particulary comprehensive and accurately measured. Secondly, we had access to individual level data from primary healthcare as well as inpatient and outpatient specialist healthcare. This is of importance when studying the diagnosis of PCC, since we have previously shown that most (>85%) patients with PCC in Sweden received their diagnosis in primary healthcare.^16^ In addition, to fully account for health seeking behaviour and the potential that the PCC group is a biased group of healthcare seekers, the number of healthcare contacts in 2019 was included as a confounder in the full model. Futhermore, the study was population based, covering the two largest regions of Sweden (Region Stockholm and Västra Götaland, 40% of the total Swedish population). Lastly, most previously published studies investigating the protective effect of vaccination before covid-19 against PCC have not been able to account for vaccinations given after infection. By not considering these vaccinations, the total protective effect against PCC will potentially be diminished as a result of the groups becoming more similar to each other. By using survival data in combination with data on vaccinations from the national vaccination register, we were able to censor individuals at vaccinations given after the acute infection.

The limitations of the present study include that both PCC and the ICD-10 diagnosis code, U09.9, are relatively new and the code has not yet been validated in a Swedish setting. It is possible that PCC might be overdiagnosed as well as underdiagnosed, which could affect both the sensitivity and the specificity of PCC as an outcome measure. If this affects both unvaccinated and vaccinated individuals fairly equally, this would lead to a non-differential misclassification of the outcome, which on average would result in some bias towards the null. However, we cannot fully rule out the possibility that vaccinated individuals are less likely than unvaccinated individuals to receive a PCC diagnosis owing to expectations from both patients and healthcare providers about the protective effect of vaccination—although it may be less likely that this bias would increase with increasing number of vaccine doses and show the strong dose-response association in our results. A recent paper from Sweden investigating healthcare use after covid-19 among patients with the PCC diagnosis code, in comparison with controls matched on age, sex, and number of healthcare contacts before infection, showed that the PCC group had significantly more healthcare contacts after covid-19.^24^ Therefore, we believe that the specificity of the PCC diagnosis code might be good, while its sensitivity remains less clear. In addition, it is possible that vaccine effectiveness differs in patients who experience a specific symptom compared with those who experience another symptom within the PCC spectrum. However, if the protective effect of the vaccines would be valid for a few specific symptoms within the PCC spectrum only, the relatively strong effect on the PCC diagnosis we see in the present study would be less likely to occur. A few studies have also investigated the impact of vaccination on existing PCC, showing both no effect as well as alleviation and aggravation of PCC symptoms.^43^ ^44^ ^45^ The register based data used in the present study had limited data on symptoms and therefore it would be difficult to assess changes in symptoms of an already existing PCC. Furthermore, although PCC is diagnosed on a specific date, the condition and symptoms usually have been present before the date of diagnosis. Lastly, our results are based on first SARS-CoV-2 infections, whereas reinfections might represent most of the infections today. The potential impact of reinfections on the covid-19 vaccine effectiveness of PCC remains to be elucidated.

### Conclusions

This study found a strong association between receiving covid-19 vaccine doses within the primary vaccination series (ie, the first two doses and the first booster dose within the recommended schedule) before infection and a reduced risk of receiving a diagnosis of PCC. Vaccine effectiveness increased with each dose in the series given before covid-19. The results from this study highlight the importance of complete primary vaccination coverage against covid-19, not only to reduce the risk of severe acute covid-19 infection but also the burden of PCC in the population.

**What is already known on this topic**

The efficacy and effectiveness of covid-19 vaccines against SARS-CoV-2 infection and severe manifestations of acute covid-19 have been shown
The effectiveness of covid-19 vaccines on post-covid-19 condition (PCC) has been less evaluated using population based real world data

**What this study adds**

The findings suggest a strong association between receiving the first three doses of vaccine before covid-19 and a reduced risk of receiving a diagnosis of PCC
Vaccine effectiveness increased with each successive dose
These results highlight the importance of primary vaccination against covid-19 to reduce the burden of PCC in the population

## Web extra

Extra material supplied by authors

## Notes

Contributors: LL, SL, FN, and MB conceived and designed the study. All authors made substantial contributions to the acquisition (YX, AS, HL, MG, FN), analysis (LL, JM, HL, MB), or interpretation (LL, SL, YX, JM, AS, MG, HL, FN, MB) of the data. LL and MB drafted the manuscript, and all authors revised it critically for important intellectual content and gave their final approval of the version to be published and agree to be accountable for all aspects of the work in ensuring that questions related to the accuracy or integrity of any part of the work are appropriately investigated and resolved. MB is the guarantor and accepts full responsibility for the work and/or the conduct of the study, had access to the data, and controlled the decision to publish. The corresponding author attests that all listed authors meet authorship criteria and that no others meeting the criteria have been omitted.

Data sharing The data used in this study are deidentified individual level data from Swedish healthcare registers and can be obtained from the respective Swedish public data holders on the basis of ethical approval for the research in question, subject to relevant legislation, processes, and data protection.

The lead author (MB) affirms that the manuscript is an honest, accurate, and transparent account of the study being reported; that no important aspects of the study have been omitted; and that any discrepancies from the study as planned (and, if relevant, registered) have been explained.

Dissemination to participants and related patient and public communities: The research findings from this manuscript will be disseminated through, for example, presentation of our findings at scientific conferences and press releases to national and international media, as well plain language summaries available on the University of Gothenburg’s homepage.

Provenance and peer review: Not commissioned; externally peer reviewed.
