# Covid-19 vaccine effectiveness against post-covid-19 condition among 589 722 individuals in Sweden: population based cohort study

**DOI:** 10.1136/bmj.q434

**Published:** 2024-02-20

In this paper by Lundberg-Morris and colleagues (*BMJ* 2023;383:e076990, doi:10.1136/bmj-2023-076990, published 22 November 2023), two rounding errors of percentages in tables S2 and S4 and a transcription error in the percentages of the stratification of severity in table S4 occurred. These tables have been corrected in the supplementary material. The updated supplementary material now also includes the stratum specific denominator numbers, to ensure clarity about how the percentages were calculated. These errors do not change the original results or conclusions of the study.

