# Supplementary material for: Covid-19 vaccine effectiveness against post-covid-19 condition among 589 722 individuals in Sweden: population based cohort study
Source: BMJ. 2023 Nov 22;383:e076990. doi: 10.1136/bmj-2023-076990 (PMC10666099; doi:10.1136/bmj-2023-076990)

Supplementary material

Covid-19 vaccine effectiveness against post-covid-19 condition among 589,722 individuals in Sweden: population based cohort study

Lisa Lundberg-Morris, Susannah Leach, Yiyi Xu, Jari Martikainen, Ailiana Santosa, Magnus Gisslén, Huiqi Li, Fredrik Nyberg, Maria Bygdell

Supplementary tables

Table S1. The five most common combinations of the first and second dose of vaccines among individuals with the first dose during the period when the three most common vaccines were administered in Sweden (3 February 2021 until 16 August 2021). Study population including all adult (≥18 years) residents in the two largest regions of Sweden, with a first registered covid-19 infection during the study inclusion period (27 December 2020 until 9 February 2022).

First and second dose	n (%)
BNT162b2[1] and BNT162b2	155,540 (79)
mRNA-1273[2] and mRNA-1273	26,294 (13)
AZD1222[3] and BNT162b2	5,118 (2.6)
AZD1222 and AZD1222	4,753 (2.4)
BNT162b2 and mRNA-1273	2,262 (1.1)

[1] Comirnaty, Pfizer/BioNTech
[2] Spikevax, Moderna
[3] Vaxzevria, Oxford-Astra Zeneca

Table S2. Reasons for termination of follow-up in study individuals, according to vaccination status before covid-19 infection. Study population including all adult (≥18 years) residents in the two largest regions of Sweden, with a first registered covid-19 infection during the study inclusion period (27 December 2020 until 9 February 2022).

	**Not vaccinated before covid-19**<br>n = 290,030	**Vaccinated before covid-19**<br>n = 299,692
Vaccination, n (%)	200,965 (69)	167,000 (56)
Reached end of follow-up[1], n (%)	73,872 (25)	126,835 (42)
Reinfection[2], n (%)	9,613 (3.3)	3,275 (1.1)
PCC, n (%)	4,118 (1.4)	1,201 (0.4)
Death, n (%)	821 (0.3)	1,076 (0.4)
Emigration, n (%)	641 (0.2)	305 (0.1)

[1]30 November 2022.
[2]New covid-19 infection at least 90 days after covid-19 index date.

PCC=post-covid-19 condition

Table S3. Vaccine effectiveness and hazard ratios, with 95% confidence intervals, between covid-19 vaccination before infection and a diagnosis of post-covid-19 condition with severity of the covid-19 infection included in the models, overall and in separate analyses stratified by number of vaccine doses. Study population including all adult (≥18 years) residents in the two largest regions of Sweden, with a first registered covid-19 infection during the study inclusion period (27 December 2020 until 9 February 2022).

	**Total**<br>n	**PCC**<br>n (%)	**Fully adjusted**[1]<br>HR (95%CI)	**p-value**	**Vaccine effectiveness**<br>% (95%CI)
No vaccination	290,030	4,118 (1.4)	*Reference*	*Reference*	*Reference*
Any prior vaccination[2]	299,692	1,201 (0.4)	0.54 (0.50 to 0.60)	<0.001	46 (40 to 50)
**Separate stratified analyses**					
1 dose[3]	21,111	192 (0.9)	0.81 (0.70 to 0.93)	0.004	19 (7 to 30)
2 doses[3]	205,650	743 (0.4)	0.53 (0.48 to 0.59)	<0.001	47 (41 to 52)
≥3 doses[3]	72,931	266 (0.4)	0.42 (0.35 to 0.49)	<0.001	58 (51 to 65)

[1]Fully adjusted Cox proportional hazards regression model: adjusted for age, sex, dominant virus variant at the time of infection, comorbidities (diabetes, cardiovascular-, respiratory-, and psychiatric disease), number of healthcare contacts in 2019, region of birth, education level, and employment status.
[2]Including 1-5 doses.
[3]Analysis performed versus no vaccination.

PCC=post-covid-19 condition, HR=hazard ratio, CI=confidence interval

Table S4. Vaccine effectiveness and hazard ratios, with 95% confidence intervals, between covid-19 vaccination (any dose) before infection and a diagnosis of post-covid-19 condition, stratified by severity of covid-19 infection, sex, age group, period of dominant virus variant at the time of infection, and comorbidities. Study population including all adult (≥18 years) residents in the two largest regions of Sweden, with a first registered covid-19 infection during the study inclusion period (27 December 2020 until 9 February 2022).

	**Total** (vaccinated/not vaccinated) n	**PCC** n (%)		**Crude** HR (95%CI)	**Partly adjusted**[1] HR (95%CI)	**Fully adjusted**[2] HR (95%CI)	**p-value**[3]	**Vaccine effectiveness**[3] % (95%CI)
		Vaccinated before covid-19	Not vaccinated before covid-19					
Severity of Covid-19 infection								
Admitted to hospital, ICU	466 (77/389)	10 (13)	106 (27)	0.47 (0.24 to 0.90)	0.71 (0.34 to 1.48)	0.77 (0.36 to 1.63)	0.50	*NA*
Admitted to hospital, not ICU	15,788 (4,610/11,178)	197 (4.3)	1,293 (12)	0.38 (0.32 to 0.44)	0.58 (0.48 to 0.69)	0.57 (0.48 to 0.68)	<0.001	43 (32 to 52)
Not admitted to hospital	573,468 (295,005/278,463)	994 (0.3)	2,719 (1.0)	0.36 (0.33 to 0.38)	0.56 (0.50 to 0.63)	0.56 (0.50 to 0.62)	<0.001	44 (38 to 50)
Sex								
Men	275,571 (132,650/142,921)	389 (0.3)	1,675 (1.2)	0.26 (0.23 to 0.29)	0.34 (0.29 to 0.39)	0.36 (0.31 to 0.42)	<0.001	64 (58 to 69)
Women	314,151 (167,042/147,109)	812 (0.5)	2,443 (1.7)	0.30 (0.28 to 0.33)	0.46 (0.41 to 0.52)	0.46 (0.41 to 0.52)	<0.001	54 (48 to 59)

Age (years)[4]								
18-35	218,029 (99,664/118,365)	206 (0.2)	598 (0.5)	0.45 (0.38 to 0.53)	0.73 (0.58 to 0.92)	0.72 (0.57 to 0.90)	0.004	28 (10 to 43)
35-44	140,125 (73,285/66,840)	260 (0.4)	919 (1.4)	0.27 (0.23 to 0.31)	0.45 (0.37 to 0.54)	0.48 (0.39 to 0.58)	<0.001	52 (42 to 61)
45-54	116,069 (60,790/55,279)	312 (0.5)	1,118 (2.0)	0.25 (0.22 to 0.28)	0.49 (0.40 to 0.59)	0.49 (0.41 to 0.60)	<0.001	51 (40 to 59)
55-64	69,325 (37,324/32,001)	230 (0.6)	979 (3.1)	0.18 (0.15 to 0.20)	0.30 (0.24 to 0.37)	0.31 (0.25 to 0.38)	<0.001	69 (62 to 75)
≥65	46,174 (28,629/17,545)	193 (0.7)	504 (2.9)	0.22 (0.18 to 0.25)	0.43 (0.34 to 0.54)	0.45 (0.35 to 0.56)	<0.001	55 (44 to 65)
Virus variant[5]								
Pre-Alpha/Alpha	238,509 (10,799/227,710)	126 (1.2)	3,513 (1.5)	1.04 (0.87 to 1.25)	0.41 (0.34 to 0.49)	0.42 (0.35 to 0.51)	<0.001	58 (49 to 65)
Delta	90,823 (64,563/26,260)	348 (0.5)	403 (1.5)	0.44 (0.38 to 0.50)	0.31 (0.26 to 0.36)	0.32 (0.27 to 0.38)	<0.001	68 (62 to 73)
Omicron	260,390 (224,330/36,060)	727 (0.3)	202 (0.6)	0.69 (0.59 to 0.81)	0.59 (0.50 to 0.69)	0.59 (0.50 to 0.69)	<0.001	41 (31 to 50)
Comorbidities[6]								
Respiratory	46,862 (25,055/21,807)	206 (0.8)	598 (2.7)	0.30 (0.26 to 0.35)	0.45 (0.36 to 0.57)	0.45 (0.36 to 0.58)	<0.001	55 (42 to 64)
Cardiovascular	97,175 (54,491/42,684)	372 (0.7)	1,196 (2.8)	0.23 (0.21 to 0.26)	0.40 (0.34 to 0.47)	0.41 (0.35 to 0.49)	<0.001	59 (51 to 65)
Diabetes	20,837	87 (0.8)	329 (3.5)	0.20 (0.16 to 0.26)	0.29 (0.21 to 0.40)	0.29 (0.21 to 0.40)	<0.001	71 (60 to 79)

	(11,449/9,388)							
Psychiatric	168,648 (86,841/81,807)	563 (0.6)	1,753 (2.1)	0.31 (0.28 to 0.34)	0.43 (0.38 to 0.49)	0.44 (0.38 to 0.50)	<0.001	56 (50 to 62)

[1]Adjusted for age, sex, and dominant virus variant at the time of infection, apart from the stratified variable.
[2]Adjusted for age, sex, dominant virus variant at the time of infection, comorbidities (diabetes, respiratory-, cardiovascular-, and psychiatric disease), number of healthcare contacts in 2019, region of birth, education level, employment status, apart from the stratified variable.
[3]Based on the fully adjusted model.
[4] Age at study start, 27 December 2020.
[5] Covid-19 infection in periods when specific virus variants were dominating. Pre-Alpha variants predominated from study start (27 December 2020) to January 2021, followed by the Alpha VOC from February 2021 to June 2021, the Delta VOC from July 2021 to December 2021, and the Omicron VOC from January 2022 until end of inclusion (9 February 2022). Due to low number of included individuals in the pre-Alpha variant period, it was joined with the Alpha period.
[6]Diagnosis codes for respiratory disease: J40-J99, cardiovascular disease: I00-I99, diabetes: E10-E11, psychiatric disease: F00-F99. The different comorbidities are overlapping, i.e. one individual can be present in more than one stratum.

PCC=post-covid-19 condition, HR=hazard ratio, CI=confidence interval, ICU=intensive care unit, VOC=variant of concern

Table S5. Vaccine effectiveness and hazard ratios, with 95% confidence intervals, between covid-19 vaccination before infection and a diagnosis of post-covid-19 condition, stratified by time between last vaccination and covid-19 infection (above/below the median time of 126 days). Study population including all adult (≥18 years) residents in the two largest regions of Sweden, with a first registered covid-19 infection during the study inclusion period (27 December 2020 until 9 February 2022).

**Time between last vaccination and covid-19 infection ≥126 days**							
	**Total** n	**PCC** n (%)	**Crude** HR (95% CI)	**Partly adjusted**[1] HR (95% CI)	**Fully adjusted**[2] HR (95% CI)	**p-value**[3]	**Vaccine effectiveness** % (95%CI)[3]
No vaccination	290,030	4,118 (1.4)	*Reference*	*Reference*	*Reference*	*Reference*	*Reference*
Any prior vaccination[4]	151,329	555 (0.4)	0.29 (0.26 to 0.31)	0.50 (0.45 to 0.57)	0.51 (0.45 to 0.58)	<0.001	49 (42 to 55)
**Separate stratified analyses**							
1 dose[5]	3,493	23 (0.7)	0.44 (0.29 to 0.66)	0.83 (0.54 to 1.26)	0.80 (0.53 to 1.22)	0.30	*NA*
2 doses[5]	147,637	532 (0.4)	0.28 (0.26 to 0.31)	0.50 (0.44 to 0.56)	0.51 (0.45 to 0.57)	<0.001	49 (43 to 55)
≥3 doses[5]	199	0 (0)	*NA*	*NA*	*NA*	*NA*	*NA*
**Time between last vaccination and covid-19 infection <126 days**							
	**Total** n	**PCC** n (%)	**Crude** HR (95% CI)	**Partly adjusted**[1] HR (95% CI)	**Fully adjusted**[2] HR (95% CI)	**p-value**[3]	**Vaccine effectiveness** % (95%CI)[3]
No vaccination	290,030	4,118 (1.4)	*Reference*	*Reference*	*Reference*	*Reference*	*Reference*
Any prior vaccination[6]	148,363	646 (0.4)	0.30 (0.27 to 0.32)	0.37 (0.33 to 0.41)	0.37 (0.34 to 0.42)	<0.001	63 (58 to 66)
**Separate stratified analyses**							
1 dose[5]	17,618	169 (1.0)	0.95 (0.81 to 1.10)	0.78 (0.67 to 0.91)	0.79 (0.67 to 0.92)	0.003	21 (8 to 33)

2 doses[5]	58,013	211 (0.4)	0.24 (0.21 to 0.28)	0.31 (0.26 to 0.35)	0.31 (0.27 to 0.36)	<0.001	69 (64 to 73)
≥3 doses[5]	72,732	266 (0.4)	0.23 (0.20 to 0.26)	0.26 (0.22 to 0.31)	0.28 (0.23 to 0.32)	<0.001	72 (68 to 77)

[1]Partly adjusted Cox proportional hazards regression model: adjusted for age, sex, and dominant virus variant at the time of infection.
[2]Fully adjusted Cox proportional hazards regression model: adjusted for age, sex, dominant virus variant at the time of infection, comorbidities (diabetes, respiratory-, cardiovascular-, and psychiatric disease), number of healthcare contacts in 2019, region of birth, education level, and employment status.
[3]Based on the fully adjusted model.
[4] Including 1-5 doses (4 doses: n=1, 5 doses: n=1).
[5]Analysis performed versus no vaccination.
[6]Including 1-5 doses (4 doses: n=82, 5 doses: n=4).

PCC=post-covid-19 condition, HR=hazard ratio, CI=confidence interval, NA=not applicable

Table S6. Vaccine effectiveness and hazard ratios, with 95% confidence intervals, between covid-19 vaccination before infection and a diagnosis of post-covid-19 condition, stratified on two vaccine doses of the five most common combinations of vaccines before infection, when the first vaccine dose was given during the period when the three most common vaccines were administered in Sweden (3 February 2021 until 16 August 2021). Study population including all adult (≥18 years) residents in the two largest regions of Sweden, with a first registered covid-19 infection during the study inclusion period (27 December 2020 until 9 February 2022).

	**Total** n	**PCC** n (%)	**Crude** HR (95%CI)	**Partly adjusted**[1] HR (95%CI)	**Fully adjusted**[2] HR (95%CI)	**p-value**[3]	**Vaccine effectiveness** % (95%CI)[3]
No vaccination	299,030	4,118 (1.4)	*Reference*	*Reference*	*Reference*	*Reference*	*Reference*
BNT162b2[4] and BNT162b2	155,540	598 (0.4)	0.30 (0.28 to 0.33)	0.48 (0.43 to 0.53)	0.49 (0.44 to 0.54)	<0.001	51 (46 to 56)
mRNA-1273[5] and mRNA-1273	26,294	88 (0.3)	0.26 (0.21 to 0.32)	0.47 (0.38 to 0.59)	0.48 (0.38 to 0.59)	<0.001	52 (41 to 62)
AZD1222[6] and BNT162b2	5,118	43 (0.8)	0.64 (0.47 to 0.86)	0.77 (0.57 to 1.04)	0.71 (0.52 to 0.96)	0.03	29 (4 to 48)
AZD1222 and AZD1222	4,753	47 (1.0)	0.86 (0.64 to 1.15)	0.41 (0.31 to 0.55)	0.43 (0.32 to 0.58)	<0.001	57 (42 to 68)
BNT162b2 and mRNA-1273	2,262	13 (0.6)	0.42 (0.24 to 0.72)	1.04 (0.60 to 1.80)	1.07 (0.62 to 1.86)	0.81	*NA*

[1]Partly adjusted Cox proportional hazards regression model: adjusted for age, sex, and dominant virus variant at the time of infection.
[2]Fully adjusted Cox proportional hazards regression model: adjusted for age, sex, dominant virus variant at the time of infection, comorbidities (diabetes, respiratory-, cardiovascular-, and psychiatric disease), number of healthcare contacts in 2019, region of birth, education level, and employment status.
[3]Based on the fully adjusted model.
[4]Comirnaty, Pfizer/BioNTech
[5]Spikevax, Moderna
[6]Vaxzevria, Oxford-Astra Zeneca

PCC=post-covid-19 condition, HR=hazard ratio, CI=confidence interval, NA=not applicable

Table S7. Vaccine effectiveness and hazard ratios, with 95% confidence intervals, between covid-19 vaccination before infection and a diagnosis of post-covid-19 condition, restricting the vaccinated population to a requirement of more than 14 days between vaccination and covid-19 infection. Study population including all adult (≥18 years) residents in the two largest regions of Sweden, with a first registered covid-19 infection during the study inclusion period (27 December 2020 until 9 February 2022).

	**Total** n	**PCC** n (%)	**Crude** HR (95%CI)	**Partly adjusted**[1] HR (95%CI)	**Fully adjusted**[2] HR (95% CI)	**p-value**[3]	**Vaccine effectiveness** % (95%CI)[3]
No vaccination	290,030	4,118 (1.4)	*Reference*	*Reference*	*Reference*	*Reference*	*Reference*
Any prior vaccination[4]	265,299	1,021 (0.4)	0.28 (0.26 to 0.30)	0.40 (0.36 to 0.44)	0.40 (0.37 to 0.45)	<0.001	60 (55 to 63)
**Separate stratified analyses**							
1 dose[5]	13,877	107 (0.8)	0.62 (0.51 to 0.76)	0.72 (0.59 to 0.88)	0.71 (0.58 to 0.86)	0.001	29 (14 to 42)
2 doses[5]	202,526	729 (0.4)	0.27 (0.25 to 0.29)	0.42 (0.38 to 0.46)	0.42 (0.38 to 0.47)	<0.001	58 (53 to 62)
≥3 doses[5]	48,896	185 (0.4)	0.25 (0.21 to 0.29)	0.26 (0.22 to 0.31)	0.27 (0.23 to 0.33)	<0.001	73 (67 to 77)

[1]Partly adjusted Cox proportional hazards regression model: adjusted for age, sex, and dominant virus variant at the time of infection.
[2]Adjusted Cox proportional hazards regression model: adjusted for age, sex, dominant virus variant at the time of infection, comorbidities (diabetes, respiratory-, cardiovascular-, and psychiatric disease), number of healthcare contacts in 2019, region of birth, education level, and employment status.
[3]Based on the fully adjusted model.
[4]Including 1-5 doses (4 doses: n=66, 5 doses: n=5).
[5]Analysis performed versus no vaccination.

PCC=post-covid-19 condition, HR=hazard ratio, CI=confidence interval

Table S8. Vaccine effectiveness and hazard ratios, with 95% confidence intervals, between covid-19 vaccination before infection and a diagnosis of post-covid-19 condition, overall and in separate analyses stratified by number of doses, with a requirement of 90 days between covid-19 infection and post-covid-19 condition. Study population including all adult (≥18 years) residents in the two largest regions of Sweden, with a first registered covid-19 infection during the study inclusion period (27 December 2020 until 9 February 2022).

	**Total** n	**PCC** n (%)	**Crude** HR (95%CI)	**Partly adjusted**[1] HR (95%CI)	**Fully adjusted**[2] HR (95% CI)	**p-value**[3]	**Vaccine effectiveness** % (95%CI)[3]
No vaccination	214,386	1,601 (0.8)	*Reference*	*Reference*	*Reference*	*Reference*	*Reference*
Any prior vaccination[4]	206,894	515 (0.3)	0.30 (0.27 to 0.33)	0.43 (0.38 to 0.49)	0.41 (0.36 to 0.47)	<0.001	59 (53 to 64)
**Separate stratified analyses**							
1 dose[5]	9,205	60 (0.7)	0.79 (0.61 to 1.03)	0.98 (0.76 to 1.28)	0.97 (0.74 to 1.26)	0.79	*NA*
2 doses[5]	132,481	326 (0.3)	0.30 (0.26 to 0.34)	0.45 (0.39 to 0.51)	0.42 (0.37 to 0.49)	<0.001	58 (51 to 63)
≥3 doses[5]	65,208	129 (0.2)	0.24 (0.20 to 0.28)	0.30 (0.24 to 0.37)	0.28 (0.22 to 0.35)	<0.001	72 (65 to 78)

[1]Partly adjusted Cox proportional hazards regression model: adjusted for age, sex, and dominant virus variant at the time of infection.
[2]Fully adjusted Cox proportional hazards regression model: adjusted for age, sex, dominant virus variant at the time of infection, comorbidities (diabetes, respiratory-, cardiovascular-, and psychiatric disease), number of healthcare contacts in 2019, region of birth, education level, and employment status.
[3]Based on the fully adjusted model.
[4]Including 1-5 doses.
[5]Analysis performed versus no vaccination.

PCC=post-covid-19 condition, HR=hazard ratio, CI=confidence interval, NA=not applicable

Table S9. Vaccine effectiveness and hazard ratios, with 95% confidence intervals, between covid-19 vaccination before infection and a diagnosis of post-covid-19 condition, restricted to those who had received two or three vaccine doses before infection. Study population including all adult (≥18 years) residents in the two largest regions of Sweden, with a first registered covid-19 infection during the study inclusion period (27 December 2020 until 9 February 2022).

	**Total** n	**PCC** n (%)	**Crude** HR (95%CI)	**Partly adjusted**[1] HR (95%CI)	**Fully adjusted**[2] HR (95% CI)	**p-value**[3]	**Vaccine effectiveness** % (95%CI)[3]
No vaccination	290,030	4,118 (1.4)	*Reference*	*Reference*	*Reference*	*Reference*	*Reference*
2 or 3 doses	278,493	1009 (0.4)	0.26 (0.24 to 0.28)	0.35 (0.32 to 0.39)	0.36 (0.32 to 0.40)	<0.001	64 (60 to 68)

[1]Partly adjusted Cox proportional hazards regression model: adjusted for age, sex, and dominant virus variant at the time of infection.
[2]Fully adjusted Cox proportional hazards regression model: adjusted for age, sex, dominant virus variant at the time of infection, comorbidities (diabetes, respiratory-, cardiovascular-, and psychiatric disease), number of healthcare contacts in 2019, region of birth, education level, and employment status.
[3]Based on the fully adjusted model.

PCC=post-covid-19 condition, HR=hazard ratio, CI=confidence interval

Supplementary figures

Figure S1. Cumulative number of first vaccine doses given before covid-19 infection, for the three most common vaccines administered in Sweden. Study population including all adult (≥18 years) residents in the two largest regions of Sweden, with a first registered covid-19 infection during the study inclusion period (27 December 2020 until 9 February 2022).

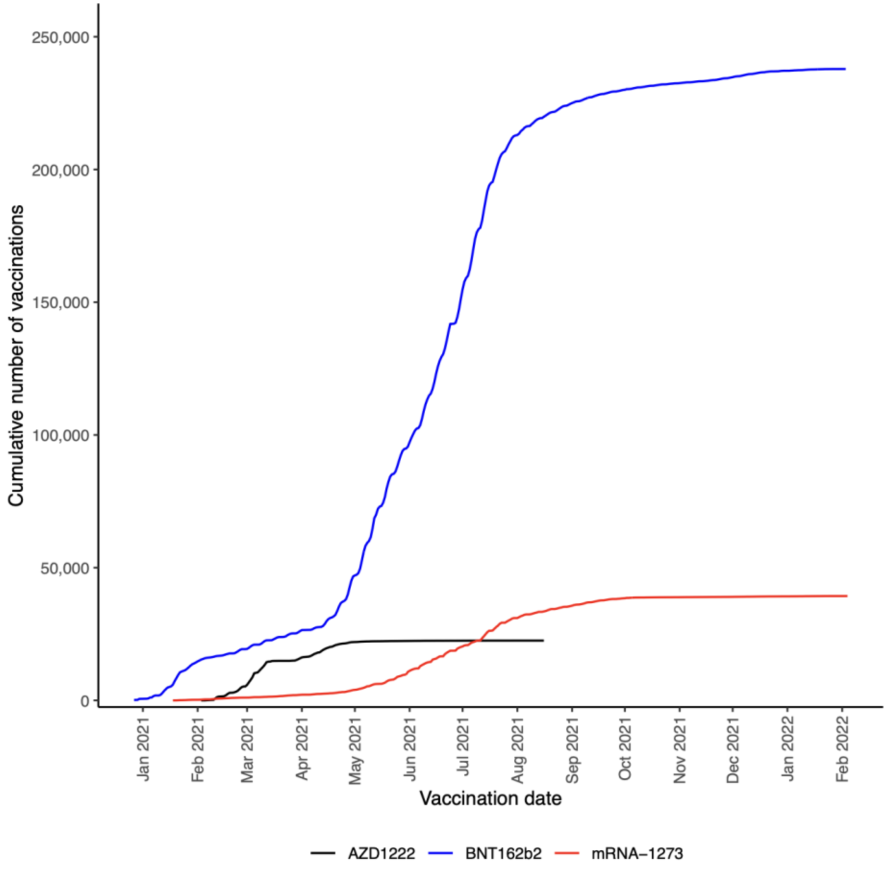

Supplement: Supplementary file 1 — Supplementary information: Additional tables S1-S9 and figure S1 [file lunl076990.ww.pdf]
